# Menopausal hormone therapy in relation to breast cancer characteristics and prognosis: a cohort study

**DOI:** 10.1186/bcr2145

**Published:** 2008-09-19

**Authors:** Lena U Rosenberg, Fredrik Granath, Paul W Dickman, Kristjana Einarsdóttir, Sara Wedrén, Ingemar Persson, Per Hall

**Affiliations:** 1 Department of Medical Epidemiology and Biostatistics, Box 281, Karolinska Institutet, SE-171 77 Stockholm, Sweden; 2 Department of Clinical Sciences, Danderyd's Hospital, Karolinska Institutet, SE-182 88 Stockholm, Sweden; 3 Department of Medicine, Unit of Clinical Epidemiology, Karolinska Institutet, SE-171 76 Stockholm, Sweden; 4 Institute of Environmental Medicine, Unit of Cardiovascular Epidemiology, 171 77 Stockholm, Sweden

## Abstract

**Introduction:**

Menopausal hormone therapy has been reported to increase the risk of certain subtypes of breast cancer and to be associated with a favorable survival. These associations could either be due to an increased mammographic surveillance or to a biological effect. We assessed these associations in a Swedish cohort of postmenopausal breast cancer patients holding information on mammographic examinations, menopausal hormone therapy use, other breast cancer risk factors, and cancer treatment.

**Methods:**

We analyzed 2,660 postmenopausal women aged 50 to 74 years, diagnosed with invasive breast cancer in 1993 to 1995 and followed until the end of 2003 (median follow-up, 9 years and 3 months). We assessed the influence of hormone therapy before diagnosis on tumor characteristics and breast cancer-specific survival. We analyzed hormone therapy before diagnosis by regimen (estrogen–progestin therapy or estrogen alone therapy), recency (current or past), and duration of use (<5 years or ≥ 5 years).

**Results:**

Current use, but not past use, compared with never use of hormone therapy before diagnosis seemed to be associated with tumors of low grade and with improved breast cancer-specific survival. The associations were stronger with longer duration, but did not vary significantly by regimen. The favorable survival among current users of hormone therapy was only partly explained by differences in available tumor characteristics and mammographic surveillance.

**Conclusions:**

We conclude that current menopausal hormone therapy, especially long term, is associated with favorable tumor characteristics and survival.

## Introduction

Menopausal hormone therapy (MHT) increases the risk of breast cancer. The risk increase is detectable after only a few years of therapy, and disappears within 5 years after discontinuation [1]. Adding progestin to estrogen therapy seems to further elevate the risk [1].

How MHT influences breast tumor characteristics is less well studied, and the results are conflicting. The only randomized controlled trial comparing estrogen–progestin therapy with placebo therapy found women in the treated arm to have slightly larger tumors with a higher proportion of lymph node involvement compared with those in the untreated arm [2]. In contrast, most observational studies either report no influence of or a favorable effect of MHT on prognostic variables such as tumor size, lymph node involvement, tumor grade, or receptor status [3-14].

The recent sharp decline in MHT use has, especially in the United States, been paralleled by a decline in the incidence of estrogen receptor (ER)-positive tumors in women aged 50 to 69 years, supporting a link between MHT and tumors of certain characteristics [15]. Most, but not all, reports on MHT use and breast cancer prognosis find a favorable survival among users of MHT before diagnosis compared with nontreated women [6,7,16-20]. It has been argued that these findings could be attributed to a higher mammographic surveillance in MHT users, which might lead to earlier diagnosis and thereby better tumor characteristics and survival [2].

To further elucidate the influence of MHT before diagnosis on tumor characteristics and breast cancer survival, we have studied 2,660 postmenopausal women with information on mammographic examinations before diagnosis and other possible confounding factors.

## Materials and methods

We used information from cases in a population-based case–control study performed in Sweden from 1993 to 1995 [21]. Briefly, all women born in Sweden and aged 50 to 74 years at first diagnosis of invasive breast cancer in the Swedish Cancer Register were eligible (*n *= 3,979), of whom 84% (*n *= 3,345) participated by answering a mailed questionnaire.

The current study was approved by the seven medical ethical review boards in Sweden. One of the ethical review boards requested a renewed informed consent for this follow-up study, while no such renewal was deemed necessary by the other boards.

We retrieved information from the six Regional Cancer Registers on tumor characteristics, and found that 45% of nonparticipants compared with 32% of participants had lymph node involvement and that nonparticipants had a 2 mm larger mean tumor size compared with participants. The mean interval from diagnosis to data collection was 4.3 months.

### Data sources

Data on possible breast cancer risk factors, including detailed information on the use of MHT, were collected through a postal questionnaire [21]. We used the national registration number, unique to each Swedish citizen, to retrieve the correct patient records and register information. We collected information on primary surgery, adjuvant treatment (endocrine therapy, chemotherapy, and radiotherapy), and tumor characteristics from surgical and oncological patient records throughout Sweden. For 35 women we did not find any patient records, and in those cases information on tumor characteristics was collected from the six Regional Cancer Registers in Sweden.

We visited 66 of the 68 units performing mammographies in Sweden, and collected information on the date and reason for the mammographies (screening or referral) performed within 5 years before diagnosis, excluding 3 months just before diagnosis to avoid registering diagnostic examinations. In the questionnaire, participants reported how many mammographies they had undergone within 1 year and 5 years, respectively, before diagnosis. This questionnaire information was used to identify cases where we failed to retrieve information from mammographic units.

We collected information on emigrations from the Swedish National Population Registry, and the date and cause of death until 31 December 2003 from the Swedish Causes of Death Registry. The latter registry covers all residents in Sweden with essentially no missing deaths, and has been shown to correctly classify 98% of breast cancer deaths [22]. The follow-up is thus virtually complete.

### Exclusions

Owing to recommendations from one ethical review board, written informed consent was sought in that region before collecting patient record data, and 67 women did not provide informed consent. In addition, we excluded 152 women with previous cancer (except nonmelanoma skin cancer), 75 cases with noninvasive breast cancer, two cases with a cancer diagnosis other than breast cancer, and 24 cases diagnosed before or after the inclusion period. Menopause was defined as the age at last menstrual period or age at bilateral oophorectomy, if it occurred 1 year or more prior to data collection. Premenopausal women (*n *= 177) as well as women below the age of 55 years with unknown age at menopause (*n *= 188) were excluded. Thus, 2,660 cases were included in the analyses.

### Classifications

Recent mammography was defined as mammography within 2 years and 2 months before diagnosis (yes/no), in order to cover the normal 2-year interval of mammographic screening plus a 2-month delay. Grade was classified according to the Nottingham histologic grade or the Bloom–Richardson scale into three groups [23]. Tumors were considered ER-positive or progesterone receptor (PR)-positive if they contained ≥ 0.05 fmol receptor/μg DNA or ≥ 10 fmol receptor/mg protein.

MHT before diagnosis was categorized into four regimens: use of estrogen (96% estradiol) in combination with progestin; estrogen alone (71% estradiol, 25% conjugated estrogens, 4% other); oral estriol; or local estrogen. We further classified MHT by duration of use (<5 years, ≥ 5 years) and by recency of use (current if <6 months, and past if ≥ 6 months between last use and diagnosis). Too few women reported use of progestin alone to be analyzed separately, and were therefore excluded from the analyses (three current users and 53 past users). Oral estriol and local estrogen were not associated with either tumor characteristics or survival (data not shown) and were therefore not defined as MHT in the analyses. Consequently, women who had only used these compounds were considered unexposed to MHT in the subsequent analyses.

Adjuvant chemotherapy and endocrine therapy (mainly tamoxifen) was classified as yes or no.

### Statistical analyses

Follow-up started at the day of diagnosis, defined as first cytological or histopathological report of cancer. The outcome was death from breast cancer (codes 174.9 and 50.9 according to the ninth and tenth revisions of international classification of diseases, respectively). The end of follow-up was defined as the date of death, the date of emigration, or 31 December 2003, whichever came first. During 21,938 person-years of follow-up, one woman emigrated, 414 women died from breast cancer, and 269 women died from other causes.

We calculated breast cancer mortality rates by background and tumor characteristics as the number of breast cancer deaths per 100 person-years. Chi-square tests were performed to detect different distributions of tumor characteristics in relation to MHT use. We used polytomous multiple logistic regression to estimate odds ratios with 95% confidence intervals (CIs) for the associations between duration of current MHT and tumor characteristics. This was done in a case-only approach – where, for each tumor characteristic, one tumor group (for instance, tumor size ≤ 10 mm) was used as the control group, and tumor sizes of 11 to 20 mm, 21 to 50 mm, and >50 mm were the outcome groups. MHT was the exposure, and never use of MHT was the reference group. Age at diagnosis and recent mammography were included as covariates.

In addition, we repeated the logistic regressions restricted to women with a recent mammography. We used the Kaplan–Meier method for assessing cause-specific survival distributions in relation to use of MHT. We compared cause-specific mortality between categories of MHT users in relation to never users with the Cox proportional hazards model. Potential confounders were recent mammography, socioeconomic status, age at first birth, parity, body mass index (kg/m^2^), height, recent alcohol intake, recent smoking, age at menopause, age at menarche, benign breast disease, and family history. If the factor was associated with breast cancer mortality, crude and stratified Cox models were compared. Tumor characteristics and treatment are intermediates between MHT exposure and breast cancer survival, and were thus included in the Cox models to assess the extent to which they could explain the observed associations. In addition, we restricted the final Cox models to women with a recent mammography.

We tested the proportional hazards assumption of duration ≥ 5 years of current MHT versus no MHT by dividing the follow-up time into >5 years or <5 years after diagnosis.

We used the SAS Statistical Software, version 9.1 (SAS Institute Inc., Cary, NC, USA) for all analyses.

## Results

The median follow-up time was 9 years and 3 months (range, 4 months to 10 years and 3 months). Breast cancer mortality rates in relation to different background and tumor characteristics are presented in Table 1. Mortality rates were slightly higher in the highest and lowest age groups. Mortality rates did not vary by socioeconomic status, but varied as expected with the tumor characteristics. Women who had undergone a recent mammography had a slightly lower mortality rate compared with other women (Table 1).

**Table 1:** Background and tumor characteristics in relation to breast cancer mortality

	Breast cancer deaths	Persons	Mortality rate^a^
Total	414	2,660	1.9
Age at diagnosis			
50 to 54 years	61	345	2.1
55 to 59 years	84	578	1.7
60 to 64 years	89	570	1.9
65 to 69 years	96	661	1.8
70 to 74 years	84	506	2.1
Socioeconomic status^b^			
Low	209	1,335	1.9
High	201	1,308	1.9
Missing	4	17	3.0
Recent mammography			
Yes	205	1,502	1.6
No	149	895	2.0
Missing	60	263	3.0
Tumor size			
≤ 10 mm	33	728	0.5
11 to 20 mm	138	1,153	1.4
21 to 50 mm	196	689	3.8
>50 mm	38	70	9.8
Missing	9	20	7.6
Lymph node involvement			
0 lymph nodes	128	1,750	0.8
1 to 3 lymph nodes	114	546	2.6
>3 lymph nodes	147	269	9.4
Missing	25	95	4.0
Grade			
1	9	272	0.4
2	73	742	1.1
3	199	764	3.4
Missing	128	882	1.8
Histology			
Ductal	301	1,901	1.9
Lobular	43	309	1.7
Other	60	415	1.7
Missing	10	35	4.3
Estrogen receptor (ER), progesterone receptor (PR) status			
ER^-^PR^-^	100	336	4.2
ER^+^PR^-^	59	286	2.6
ER^-^PR^+^	21	72	3.9
ER^+^PR^+^	148	1172	1.5
Missing	86	794	1.3

^a^Breast cancer deaths per 100 person-years. ^b^Low level includes blue-collar workers and low-level white-collar workers, whereas high level includes all other categories.

No difference in the distribution of age or of tumor characteristics was noted when comparing past use and never use of MHT. Past users were more often of high socioeconomic status and seemed to have been through a recent mammography more often (Table 2). Current users of MHT were younger at date of diagnosis, were more often of high socioeconomic status, and more often had undergone a recent mammography. Current use of MHT was not associated with tumor size or lymph node involvement. On the other hand, low-grade tumors, tumors of nonductal histological type, ER^+^PR^+ ^tumors, and ER^-^PR^+ ^tumors were more often found among current MHT users compared with never MHT users (Table 2).

**Table 2:** Distribution of background, tumor characteristics, and recent mammography in relation to use of menopausal hormone therapy

	Never use	Past use	Current use	Missing
Total	1,788	253	523	96
Age at diagnosis				
50 to 54 years	154 (9)	23 (9)	148 (28)	20
55 to 59 years	320 (18)	45 (18)	181 (35)	32
60 to 64 years	381 (21)	54 (21)	116 (22)	19
65 to 69 years	521 (29)	70 (28)	55 (11)	15
70 to 74 years	412 (23)	61 (24)	23 (4)	10
*P *value^a^		0.99	<0.0001	
Socioeconomic status^b^				
Low	961 (54)	120 (47)	197 (38)	57
High	811 (46)	133 (53)	325 (62)	39
Missing	16	0	1	0
*P *value^a^		0.042	<0.0001	
Recent mammography				
Yes	933 (59)	147 (64)	361 (73)	61
No	650 (41)	83 (36)	133 (27)	29
Missing	205	23	29	6
*P *value^a^		0.15	<0.0001	
Tumor size				
≤ 10 mm	488 (28)	59 (24)	151 (29)	30
11 to 20 mm	759 (43)	115 (46)	240 (46)	39
21 to 50 mm	471 (27)	72 (29)	120 (23)	26
>50 mm	56 (3)	3 (1)	10 (2)	1
Missing	14	4	2	0
*P *value^a^		0.17	0.15	
Lymph node involvement				
0 lymph nodes	1,164 (68)	164 (67)	353 (69)	69
1 to 3 lymph nodes	362 (21)	53 (22)	113 (22)	18
>3 lymph nodes	187 (11)	29 (12)	46 (9)	7
Missing	75	7	11	2
*P *value^a^		0.90	0.44	
Mean number of examined lymph nodes	10.4	10.8	10.7	
Grade				
1	164 (14)	17 (10)	78 (23)	13
2	524 (43)	65 (40)	133 (39)	20
3	520 (43)	81 (50)	130 (38)	33
Missing	580	90	182	30
*P *value^a^		0.23	0.0002	
Histology				
Ductal	1,314 (74)	180 (72)	338 (66)	69
Lobular	186 (11)	31 (12)	81 (16)	11
Other	269 (15)	38 (15)	93 (18)	15
Missing	19	4	11	1
*P *value^a^		0.64	0.0004	
Estrogen receptor (ER), progesterone receptor (PR) status				
ER^-^PR^-^	227 (18)	37 (19)	55 (15)	17
ER^+^PR^-^	200 (16)	32 (17)	40 (11)	14
ER^-^PR^+^	42 (3)	5 (3)	23 (6)	2
ER^+^PR^+^	776 (62)	116 (61)	240 (67)	40
Missing	543	63	165	23
*P *value^a^		0.91	0.0044	

Data presented as *n *(%). Past use, last use at least 6 months before diagnosis; current use, last use <6 months before diagnosis. ^a^Two-sided global chi-squared *P *value for past use and current use, respectively, compared with no use of menopausal hormone therapy. ^b^Low level includes blue-collar workers and low-level white-collar workers, whereas high level includes all other categories.

We subdivided current MHT use by regimen and duration (Table 3). Recent mammography and tumor characteristics did not vary significantly by regimen among current users of MHT. Long-term current use (≥ 5 years) compared with short-term current use (<5 years) was associated with smaller tumor size, but did not seem to differ in lymph node involvement. Long-term current users more often had tumors of low grade, of nonductal histological type, and of ER^+^PR^+ ^status compared with short-term current users (Table 3). There were fewer patients with a recent mammography among long-term current users compared with short-term current users, but the difference was not significant (Table 3).

**Table 3:** Distribution of tumor characteristics, and recent mammography among current users of menopausal hormone therapy by type and duration

	Therapy			Duration		
		
	Estrogen–progestin	Estrogen alone	*P *value^a^	<5 years	≥ 5 years	*P *value^a^
Total	422	92		238	284	
Recent mammography						
Yes	301 (75)	55 (66)	0.11	171 (76)	189 (70)	0.13
No	102 (25)	28 (34)		53 (24)	80 (30)	
Tumor size						
≤ 10 mm	125 (30)	23 (25)		61 (26)	90 (32)	
11 to 20 mm	186 (44)	51 (56)	0.19	101 (43)	139 (49)	0.010
21 to 50 mm	102 (24)	15 (16)		70 (30)	49 (17)	
>50 mm	8 (2)	2 (2)		5 (2)	5 (2)	
Lymph node involvement						
0 lymph nodes	289 (70)	60 (67)		162 (69)	191 (69)	
1 to 3 lymph nodes	86 (21)	24 (27)	0.38	47 (20)	65 (23)	0.36
>3 lymph nodes	39 (9)	6 (7)		25 (11)	21 (8)	
Grade						
1	69 (25)	10 (15)		21 (13)	57 (31)	
2	99 (37)	32 (48)	0.11	57 (36)	76 (42)	<0.0001
3	103(38)	24 (36)		80 (51)	50 (27)	
Histology						
Ductal	272 (66)	61 (68)		17 (74)	166 (60)	
Lobular	69 (17)	10 (11)	0.37	34 (15)	46 (17)	0.0007
Other	73 (18)	19 (21)		27 (12)	66 (24)	
Estrogen receptor (ER), progesterone receptor (PR) status						
ER^-^PR^-^	45 (15)	9 (16)		34 (20)	21 (11)	
ER^+^PR^-^	35 (12)	5 (9)	0.93	25 (15)	15 (8)	0.0062
ER^-^PR^+^	19 (6)	4 (7)		13 (8)	10 (5)	
ER^+^PR^+^	196 (66)	39 (68)		99 (58)	140 (75)	

Data presented as *n *(%). ^a^Global chi-squared *P *value for type and duration, respectively.

We performed polytomous logistic regression with one group in each tumor characteristic as the control group and the other groups as the case groups, comparing long-term and short-term current use with never use and adjusting for age at diagnosis and recent mammography (Table 4). The odds ratios were only marginally influenced by age at diagnosis and mammography use, and the results were thus essentially the same as those presented in Table 3. The odds ratio of being diagnosed with a grade 3 tumor instead of a grade 1 tumor was 0.3 (95% CI, 0.2 to 0.5) for long-term current use compared with never users of MHT. As current use of MHT was related to recent mammography, and the use of mammography is related to breast cancer survival, we repeated the regression analyses restricted to women with a recent mammography, and the results were essentially unaltered apart from the estimates among long-term current use compared with never use regarding tumor size, where the point estimates for tumor sizes 21 to 50 mm and >50 mm were closer to unity, and their CIs were nonsignificant (data not shown).

**Table 4:** Duration of current use relative to never use of menopausal hormone therapy and risk of breast tumor characteristics

	<5 years current MHT		≥ 5 years current MHT	
		
	Crude	Adjusted^a^	Crude	Adjusted^a^
Tumor size				
≤ 10 mm	Reference	Reference	Reference	Reference
11 to 20 mm	1.1 (0.8 to 1.6)	1.1 (0.8 to 1.7)	1.0 (0.7 to 1.3)	1.0 (0.8 to 1.4)
21 to 50 mm	1.4 (0.9 to 2.0)	1.2 (0.8 to 1.9)	0.6 (0.4 to 0.9)	0.6 (0.4 to 0.9)
>50 mm	1.0 (0.4 to 2.6)	0.8 (0.3 to 2.3)	0.6 (0.2 to 1.6)	0.7 (0.2 to 1.7)
Lymph node involvement				
0 lymph nodes	Reference	Reference	Reference	Reference
1 to 3 lymph nodes	1.0 (0.7 to 1.4)	0.8 (0.5 to 1.2)	1.2 (0.9 to 1.7)	1.1 (0.8 to 1.6)
>3 lymph nodes	0.9 (0.6 to 1.5)	0.8 (0.5 to 1.4)	0.7 (0.4 to 1.1)	0.6 (0.4 to 1.1)
Grade				
1	Reference	Reference	Reference	Reference
2	0.9 (0.5 to 1.6)	1.3 (0.7 to 2.4)	0.4 (0.3 to 0.7)	0.5 (0.3 to 0.7)
3	1.3 (0.8 to 2.3)	1.5 (0.8 to 2.7)	0.3 (0.2 to 0.5)	0.3 (0.2 to 0.5)
Histology				
Ductal	Reference	Reference	Reference	Reference
Lobular	1.4 (1.0 to 2.2)	1.5 (1.0 to 2.5)	1.9 (1.3 to 2.8)	2.0 (1.4 to 2.9)
Other	0.8 (0.5 to 1.2)	0.9 (0.5 to 1.4)	2.0 (1.4 to 2.7)	2.0 (1.4 to 2.8)
Estrogen receptor (ER), progesterone receptor (PR) status				
ER^-^PR^-^	Reference	Reference	Reference	Reference
ER^+^PR^-^	0.9 (0.5 to 1.5)	1.2 (0.6 to 2.4)	0.9 (0.4 to 1.8)	1.0 (0.5 to 2.1)
ER^-^PR^+^	1.9 (0.9 to 4.0)	1.5 (0.6 to 3.7)	2.6 (1.1 to 6.0)	2.8 (1.2 to 6.7)
ER^+^PR^+^	0.9 (0.6 to 1.4)	1.1 (0.7 to 1.8)	2.0 (1.2 to 3.3)	2.1 (1.3 to 3.6)

Data presented as the odds ratio (95% confidence interval) relative to never users of menopausal hormone therapy (MHT). For each tumor characteristic, the first row is the control group, and the other groups are the case groups. Never use of MHT is the reference group, and <5 years or > 5 years of current MHT are the exposure groups. ^a^Adjusted for recent mammography (yes/no) and age at diagnosis (5-year categories).

**Figure 1:**
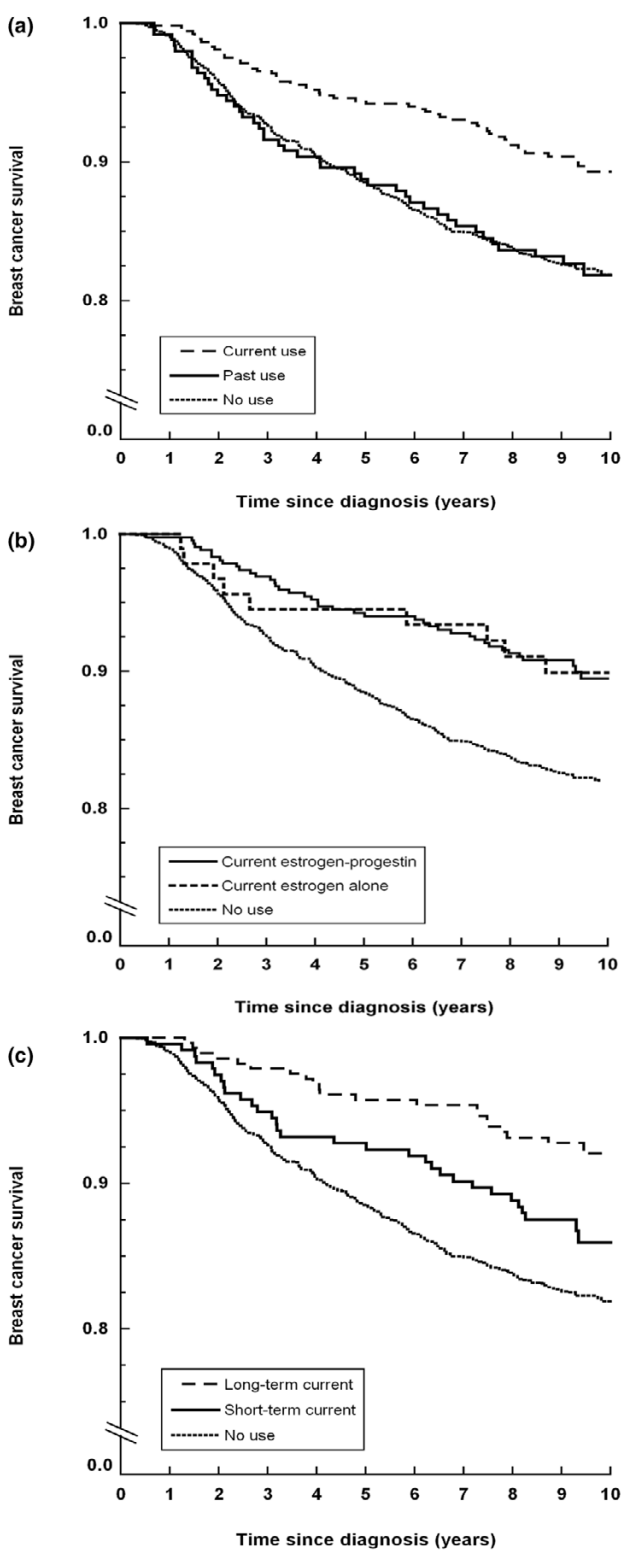
**Kaplan–Meier plots of breast cancer-specific survival in relation to use of menopausal hormone therapy**. **(a) **No use, current use, or past use. **(b) **No use or current use by regimen. **(c) **No use or current use by duration.

Figure 1 shows Kaplan–Meier plots for never use, past use, and current use of MHT (Figure 1a) as well as by regimen (Figure 1b) and by duration (Figure 1c). Almost 20% of past users and never users had died from breast cancer 10 years after diagnosis, compared with approximately 10% of current users (Figure 1a). Breast cancer survival did not differ by regimen of current use (Figure 1b). When we compared short-term and long-term current use, long-term users had a more favorable breast cancer survival – particularly so during the first 3 years after diagnosis (Figure 1c).

The hazard ratio for dying from breast cancer for current MHT use compared with never MHT use was 0.57 (95% CI, 0.41 to 0.79; Table 5). The favorable survival among current MHT users seemed to be present regardless of the receptor status: hazard ratio in ER^+^PR^+ ^tumors, 0.40 (95% CI, 0.22 to 0.72); and hazard ratio in ER^-^PR-tumors, 0.56 (95% CI, 0.26 to 1.19) (data not shown). Adjusting for other breast cancer risk factors did not affect the survival estimate (data not shown). Restricting the analysis to women with a recent mammography did not alter the estimate compared with adjusting for recent mammography (data not shown). Past MHT use did not seem to influence breast cancer survival (Table 5).

**Table 5:** Hazard ratios of dying from breast cancer in relation to use of menopausal hormone therapy

	Breast cancer deaths/exposed (*n*)	Mortality rate^a^	Hazard ratio (95% confidence interval)^b^	Hazard ratio (95% confidence interval)^c^
Never use	230/1,491	1.9	1.0 (reference)	1.0 (reference)
Current use^d^	46/477	1.1	0.55 (0.40 to 0.77)	0.63 (0.42 to 0.95)
By duration				
<5 years	28/216	1.5	0.73 (0.48 to 1.13)	0.76 (0.45 to 1.27)
≥ 5 years	18/260	0.8	0.42 (0.26 to 0.68)	0.52 (0.29 to 0.93)
By regimen				
Estrogen^-^progestin	37/391	1.1	0.54 (0.37 to 0.77)	0.59 (0.38 to 0.91)
Estrogen alone	8/80	1.1	0.59 (0.29 to 1.20)	0.78 (0.34 to 1.80)
Past use^e^	34/219	1.9	1.01 (0.71 to 1.45)	1.03 (0.68 to 1.54)
By duration				
<5 years	25/155	1.9	1.04 (0.69 to 1.58)	1.02 (0.64 to 1.63)
≥ 5 years	9/64	1.7	0.94 (0.48 to 1.83)	1.02 (0.48 to 2.17)

Observations with missing information on menopausal hormone therapy, recent mammography, tumor size, or lymph node involvement excluded. ^a^Breast cancer deaths per 100 person-years. ^b^Adjusted for age at diagnosis (5-year categories), recent mammography (yes/no). ^c^Adjusted for age at diagnosis (5-year categories), recent mammography (yes/no), adjuvant endocrine therapy (yes/no) and adjuvant chemotherapy (yes/no), tumor size, and lymph node involvement. ^d^Last use <6 months before diagnosis. ^e^Last use at least 6 months before diagnosis.

Long-term current users seemed to be the group with the most favorable survival. The hazard ratio was 0.41 (95% CI, 0.25 to 0.67) for long-term current users and was 0.78 (95% CI, 0.51 to 1.19) for short-term current users compared with never MHT use (Table 5). The regimen of use had no significant influence on the survival.

When we included tumor size and lymph node involvement in the models, the estimates for various aspects of current MHT use were slightly attenuated (Table 5). Owing to a large proportion of missing information on grade and receptor status, we were not able to include these characteristics simultaneously in the models. Adding either the ERPR status or grade to models with tumor size and lymph node involvement did not affect the survival estimates more than marginally (data not shown).

The hazard ratio for the first 5 years after diagnosis was 0.48 (95% CI, 0.25 to 0.93), and was 0.71 (95% CI, 0.39 to 1.30) for the period thereafter, when comparing current users with never users and adjusting for age at diagnosis, recent mammography, treatment, tumor size, and lymph node involvement. The proportional hazards assumption was thus not significantly rejected, but the favorable effect of MHT seemed to be larger in the first 5 years after diagnosis than later during follow-up. Excluding one woman with current use and 32 women with never use of MHT who had distant metastases at diagnosis did not change the survival estimates (data not shown).

## Discussion

Current use, but not past use, of MHT was associated with tumor grade, histological type, and hormone receptor status, but not associated with tumor size or lymph node status. The breast cancer-specific survival was more favorable among current users compared with never users of MHT after adjusting for age and recent mammography. Available tumor characteristics explained part of this observation. The protective effect seemed to be more pronounced in the first 5 years after diagnosis. The association between current MHT use and tumor characteristics and breast cancer-specific survival was more pronounced among long-term users than short-term users, but did not seem to vary according to the regimen of MHT.

Tumor progression from *in situ *tumor to metastatic tumor has, according to the multistep view, been thought to be paralleled by sequentially accumulating genetic alterations. According to this view, tumors would progress from ER-positive to ER-negative and from low grade to high grade during tumor progression. An alternative hypothesis is that the tumor phenotype is determined early during carcinogenesis and remains stable during the clinical phase [24]. Gene expression studies have found breast tumors clustering in a few groups related to ER status and grade, rather than a continuum of patterns [24]. ER-positive tumors versus ER-negative tumors, and low-grade tumors versus high-grade tumors, also have mutually exclusive mutations – making transition to ER-negative tumors and to high-grade tumors, respectively, during late tumor progression less likely [25]. One study compared tumor characteristics in tumors diagnosed at first screening and subsequent screening rounds, and found that tumor size and lymph node involvement was more advanced in the first round, whereas tumor grade did not differ between rounds [26]. In the light of these findings, it seems plausible that receptor status and tumor grade are relatively stable characteristics reflecting an established phenotype, while tumor size and lymph node involvement reflect a combination of the phenotype and age of the tumor.

After taking mammographic surveillance before diagnosis (mostly screening-based settings) into consideration, most observational studies have found little association between current MHT use and time-dependent tumor characteristics, such as tumor size and lymph node involvement [3,7-9,11-13,27,28]. Most previous studies have found an association between MHT use and prognostic factors reflecting the biology of the tumors – that is, low-grade tumors [3,5,9,10,12,14,29] – although conflicting results exist [8,13].

In the Women's Health Initiative randomized clinical trials [2,30], women treated with estrogen alone or treated with estrogen–progestin had slightly larger tumors and a higher prevalence of lymph node involvement than untreated women. For tumor grade, receptor status, and histological type, the only significant difference reported in the randomized trials was that women treated with estrogen alone were more likely to have lobular tumors than ductal tumors. The discrepancy between these results and those from observational studies is puzzling. It could be that observational studies finding similar or smaller tumors among MHT users have not fully corrected for lead-time bias, while the absence of association between MHT use and low-grade tumors in the randomized trials might be due to low numbers, the 40% noncompliance, and possibly the high proportion of obese women.

Seven studies [6,7,16,18-20,31] out of the eight previous studies [6,7,16-20,31] support our finding of a favorable survival after breast cancer among MHT users compared with nonusers. Two screening-based studies found reduced breast cancer-specific mortality after adjustment for tumor size and lymph node involvement [7,20], and one of them also for tumor grade. Two studies examined duration of use, but, in contrast to our findings, they found no association [19,20]. The only other study to examine the regimen of MHT found a nonsignificantly lower mortality with estrogen–progestin use compared with estrogen alone [16]. All studies that have evaluated recency of use, including ours, have found the improved survival to be mainly confined to current use [7,16,18].

The favorable breast cancer survival among current MHT users was more pronounced in the first 5 years after diagnosis than thereafter. This is in line with the finding that more aggressive breast cancer cases have a peak mortality after around 2 years, while less aggressive breast cancer cases have a low but constant mortality for >10 years after diagnosis [32]. If our follow-up had lasted longer, therefore, the expected survival benefit due to MHT would have diminished.

As MHT increases the risk of breast cancer after only a few years, and this risk disappears shortly after ceasing, MHT probably acts as a late-stage promoter of breast cancer. The epidemiological findings of an association between current MHT use and low-grade, receptor-positive tumors indicate that MHT may promote preclinical tumors with less malignant tumor characteristics. Consequently, we think that current MHT use affects survival through biological effects on the tumor. We cannot, however, rule out an influence of a healthy women effect, or of lead-time bias. The highest risks of getting breast cancer are found among long-term users, current users, or estrogen–progestin users [1]. If MHT influences the tumor phenotype, these subgroups are likely to be most affected, and our findings of favorable tumor characteristics and improved survival in these women are plausible.

The use of MHT has dropped since 2002 in the United States [33,34], and since 1999 in Sweden [35]. A decreased incidence of ER-positive breast cancer in women aged 50 to 69 years has been reported after 2002 [33,34], and a common belief is that this decrease is due to the decreased use of MHT. As we and other workers have found MHT linked to tumors of low aggressiveness, we think a possible decreased breast cancer incidence due to decreased use of MHT will be accompanied by a much smaller decrease in breast cancer mortality, which will probably not be possible to detect at the population level.

The strengths of the present study are the population-based setting with a high participation rate, the detailed information on MHT use and other breast cancer risk factors, information on tumor characteristics as well as surgical and adjuvant treatment, only one patient being lost to follow-up, and high-quality information on the cause of death [22].

There are limitations to the present study. Among the eligible cancer cases in the study, 16% did not participate. Nonparticipants had somewhat larger tumors and a higher proportion of lymph node involvement. If nonparticipants using MHT had worse tumor characteristics than participants using MHT, or if nonparticipants not using MHT had better tumor characteristics than participants not using MHT, our results would be biased – but this seems unlikely. Receptor status was assessed at seven different laboratories, and many different pathologists classified the tumor size, lymph node involvement, tumor grade, and histological type. The proportion of missing values was high for some of these variables. The misclassification due to the decentralized analyses as well as the distribution of missing values was probably not related to MHT use, and should thus be nondifferential. We lack information on MHT use after diagnosis. Women diagnosed with breast cancer were strongly recommended not to use MHT – so even if some women used MHT after diagnosis, this was rarely reported in the surgical and oncological patient records. We do not, however, believe that MHT after diagnosis could influence our results more than marginally, being a probably rare exposure with an unknown and probably small effect on survival [36]. Even though our study included more than 2,500 women diagnosed with breast cancer, the power for many of the subgroup analyses was small and precluded firm conclusions.

## Conclusion

We found that use of MHT at the time of breast cancer diagnosis was associated with lower tumor grade, lobular or other nonductal histology, positive receptor status, and with a favorable breast cancer survival. Mammographic surveillance did not explain our results. As the tumor grade and receptor status seem to be relatively stable characteristics that are not sensitive to lead-time bias, we believe that MHT induces tumors of certain phenotypes.

## Abbreviations

CI: confidence interval; ER: estrogen receptor; MHT: menopausal hormone therapy; PR: progesterone receptor.

## Competing interests

The authors declare that they have no competing interests.

## Authors' contributions

LUR participated in the study design, data collection, statistical analysis and interpretation, and drafted the manuscript. FG and PWD participated in the study design, statistical analysis, and interpretation. KE participated in the interpretation. SW participated in the study design and interpretation, and helped to draft the manuscript. IP participated in the study design and data collection. PH participated in the study design, data collection and interpretation of the data, and helped to draft the manuscript. All authors read and approved the final manuscript.
